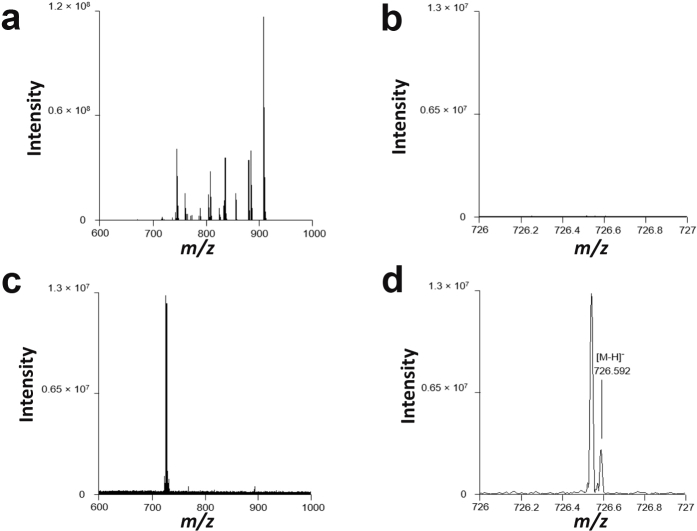# Spatial and temporal brain biodistribution of neuropathogenic sphingolipids of Krabbe disease

**DOI:** 10.1016/j.jlr.2025.100960

**Published:** 2025-12-05

**Authors:** Tingting Yan, Shih-Chang Hsueh, Salma Begum, Narayana Chelakkara Venkiteswaran, Justin Ellenburg, Boone M. Prentice, Gustavo H.B. Maegawa

**Affiliations:** 1 Department of Chemistry, University of Florida, Gainesville, FL; 2 Department of Pediatrics, Genetics and Metabolism; 3 Department of Medicine, Cardiology, Columbia University Irving Medical Center, New York, NY

**Keywords:** Globoid-cell leukodystrophy, Krabbe disease, matrix-assisted laser deabsorption/ionization imaging mass spectrometry (MALDI IMS), psychosine, galactosylceramide

## Abstract

Glycosphingolipids are essential lipids enriched in the outer leaflet of the plasma membrane, particularly those forming the myelin sheaths. Disorders impacting the glycosphingolipid metabolism cause devastating demyelinating diseases. We extend this observation by investigating the brain topographic pattern of the progressive accumulation of these glycosphingolipids throughout the lifespan of the murine model, correlating with alterations in myelin markers and astrogliosis. The reported IMS approach reveals disturbances in the brain glycosphingolipid spatial distribution and abundance, which is of utmost importance when examining the impact of neurotherapeutics targeting these cytotoxically elevated sphingolipids. Similar approaches can be applied to other sphingolipid-neurodegenerative disorders. The development of a novel imaging mass spectrometry (IMS) method provides key information on the spatial distribution and quantification of pathogenic glycosphingolipids, hexosphingosines, and monohexosylceramides across the brain of the murine model of Krabbe disease, an inherited lysosomal deficiency of galactocerebrosidase, associated with a demyelinating disorder with a broad spectrum of age of onset.

Imaging mass spectrometry is a label-free analytical technology that provides relative abundances and spatial distributions of a large variety of biomolecules, such as lipids, peptides, proteins, metabolites, and amino acids, with a high degree of molecular specificity within tissue slices (1). Each irradiated tissue area generates a mass spectrum, and sequential irradiated areas are acquired in a raster pattern across the tissue. Following the acquisition, the relative intensity and spatial localization of ions of interest can be viewed, with each individual irradiated area representing a pixel in the resulting molecular image. Matrix-assisted laser desorption/ionization imaging mass spectrometry (MALDI IMS) of lipids has been quite successful, given that lipids are abundant in specific biological tissues, particularly the nervous system (2). Besides cardiolipins and gangliosides, lipids generally have molecular masses between 200 and 1,000 Da, a readily detectable mass range of many mass spectrometry instruments. Fourier transform ion cyclotron resonance (FT-ICR) mass spectrometers are particularly well suited to these experiments, being noted for high mass resolving power and accurate mass measurements (3, 4).

Globoid-cell leukodystrophy (GLD), or Krabbe disease, is a lysosomal disorder caused by the deficiency of galactosylceramidase (Galc) secondary to biallelic pathogenic variants in the *galc* gene that result in impaired activity of the enzyme. Because of the Galc deficiency, the degradation of two galactosphingolipid substrates of Galc, galactosylceramide and galactosylsphingosine, mostly known as psychosine, is found at increased levels. The accumulation of galactosylceramide in macrophages and microglia results in the pathological hallmark of GLD: multinucleated histiocytes known as globoid cells (5). Meanwhile, psychosine is highly toxic to myelin-forming cells such as oligodendrocytes and Schwann cells in the central (CNS) and peripheral (PNS) nervous systems, respectively. Previous studies have shown that psychosine disrupts the architecture and composition of lipid rafts and activates apoptotic pathways that ultimately result in demyelination (6, 7, 8, 9, 10). Among the clinical forms, the infantile GLD is the most prevalent, known as ‘classic,’ and subdivided into an early- and late-infantile GLD with onset before or after six months of age, and progression relatively homogeneous (11, 12). More recent data indicate that infantile GLD clinical form represents ∼70% of affected patients, and 30% with a later onset from childhood to adulthood (11, 13).

While several studies have investigated levels of the primary Galc substrates, psychosine, and galactosylceramide, there is a lack of knowledge about the spatial distribution of these pathogenic lipids in the brain, mainly when investigating therapeutic interventions in research. Herein, a novel MALDI IMS method was developed to detect, with high sensitivity, the sphingolipids elevated in brain sections from the classical murine GLD model, the Twitcher mouse *Galc*^*twi/twi*^ (TWI) (14). For the first time, the hexosylsphingosines (HexSP) and monohexosylceramides (HexCer; d18:1/18:0) distribution in the brains of the TWI mice across their lifespan is reported compared to age-matched wild-type (C57BL6 controls). The correlations between the sphingolipid distribution at different ages in the murine GLD model and pathogenic processes in the CNS were also reported, along with confirmatory LC-MS/MS measurements. This specific and semi-quantitative MALDI IMS method showed that the pathogenic sphingolipids are elevated in particular brain areas in the setting of Galc deficiency, which can then help ascertain the efficacy and biodistribution of the upcoming CNS-target therapeutics for GLD.

## Materials and methods

### Experimental model

All animal experiments were performed in accordance with Columbia University Irving Medical Center IACUC-approved protocols. Twitcher mouse *Galc*^*twi/twi*^(Twi) mice were obtained as a donation from Dr Ernesto Bongarzone, PhD (University of Illinois, Chicago, IL), and a breeding colony was maintained in our laboratory. As previously reported, genotyping was performed using polymerase chain reaction (PCR) (15, 16). Twitcher and C57BL6 *Galc*^*wt/wt*^ (WT) mice were euthanized via CO_2_ asphyxiation followed by decapitation. Whole brain tissue was dissected and immediately snap-frozen in dry ice and isobutane to maintain spatial integrity and stored at −80°C.

### Brain sections and immunofluorescence staining

For the immunofluorescence, snap-frozen brain specimens were cryosectioned at 14–16 μm and mounted to indium tin oxide (ITO) coated slides (catalog# CG-81IN-S115) and regular Superfrost slides (Fisher). The ITO slides used in the MALDI IMS experiments have a sheet resistance, Rs = 30–60 Ω/sq, nominal transmittance, > 83%, nominal coating thickness, 300–600 Å, and substrate thickness of 0.7 and 1.1 mm. The immunofluorescence staining was performed using paraformaldehyde 4% for fixation over 20 min, followed by permeabilization with 0.1% Triton-X in phosphate-buffered saline (PBS) buffer, 60 min of blocking with 5% goat serum in PBS, before incubation with primary antibodies overnight (10). The slides were washed three times with PBS before loading the secondary antibody for 4 h. The slides were mounted with ProLong™ Gold (cat.#P36934). The following primary antibodies were used: rabbit anti-glial fibrillary acidic protein (GFAP; Encor, catalog#RPCA-GFAP), goat anti-myelin binding protein (MBP; Encor, catalog# GPCA-MBP), rabbit anti-neurofilament H (NLF-H, Encor# RCPA-NF-H). The following secondary antibodies were used: F(ab’)2 fragment AlexFluor 488 donkey anti-rabbit (Jackson Labs; cat.# 711-546-152); F(ab’)2 fragment AlexFluor 594 donkey anti-goat (Jackson Labs; cat.# 705-586-147). Images were captured using the Cytation 5 imaging instrument (Agilent).

### MALDI matrix application

Slides containing mounted tissue sections, stored in a −80°C freezer were removed and warmed to room temperature in a desiccator for ∼30 min before matrix application. A custom-built sublimation apparatus was used for the MALDI matrix application. A 2′,6′-dihydroxyacetophenone (DHA) matrix was used for HexSP analysis and was sublimated at 120°C for 5 min, resulting in 1.5–2.0 mg of matrix deposited onto the slide. A 1,5-diaminonaphthalene (DAN) matrix was used for HexCer analysis and was sublimated at 115°C for 7 min, resulting in 1.5–2.5 mg of matrix deposited onto the slide.

### Imaging mass spectrometry

All imaging mass spectrometry experiments were performed using a 7 T solariX Fourier transform ion cyclotron resonance (FT-ICR) mass spectrometer equipped with a dynamically harmonized ICR cell (Bruker Daltonics). A Smartbeam II Nd: YAG MALDI laser system (2 kHz, 355 nm) was operated at a repetition rate of 2 kHz. HexSP and HexCer images were acquired using a pixel spacing of 125 or 150 μm in both the x and y dimensions using a Smart Walk. External calibration was performed using spotted standards on tissue sections. Continuous accumulation of selected ions (CASI) was used to enrich for HexSP (473.00±17.5 Da) and HexCer (728.00±5 Da) mass ranges of interest, resulting in significantly improved signal intensity compared to full-scan measurements (17, 18). HexSP data were acquired from *m/z* 300 to 1,000 using a 0.3670-s time-domain transient length, resulting in a mass resolving power of ∼40,000 at *m/z* 430. HexCer data were acquired from *m/z* 250 to 1,000 using a 2.4117-s time-domain transient length, resulting in a mass resolving power ∼170,000 at *m/z* 726. All ion images were visualized without normalization using SciLS (Bruker Daltonics). Regarding HexSP detection, CASI was performed using an *m/z* window of 30 Th centered at *m/z* 460. For HexCer detection, CASI was utilized using an m/z window of 10 Th centered at *m/z* 729.

### LC-MS/MS

Cerebrum specimens from C57/BL6 Twitcher mice (*Galc*^*twi/twi*^; TWI) and age-matched C57BL6 *Galc*^*wt/wt*^ (WT) were dissected and homogenized in 9:1 (v/v) in buffer A, which consists of 0.25 M sucrose, 0.5 mM EDTA, 50 mM KCl, 25 mM KCl at pH 7.4. Afterward, protein determination was performed using BCA (ThermoFisher), and 1 mg was removed for lipid extraction in 9:1 (v/v) of methanol containing 20 ng/ml D5-psychosine as an internal standard (Avanti Polar) (19). LC-MS/MS psychosine quantification was based on a previous method (20). An Acquity UPLC HSS C18 (2.1 × 100 mm, 1.8um; Waters #186003533) on a Waters Acquity UPLC system was coupled to Waters Xevo TQS for LC-MS/MS measurements. The flow rate is 0.3 mL/min, and the temperature of the column is 40°C. The mobile phases comprised 0.1% Formic acid (mobile phase A) and acetonitrile containing 0.1% formic acid (mobile phase B). The ESI (electrospray ionization) source was operated in positive ion mode at 3 KV. The desolvation temperature was kept at 50°C, and the desolvation gas flow was 1000 L/h with cone gas at 150 L/h. Multiple reaction monitoring (MRM) transitions were used to generate fragment ions from precursor [M+H]+ ions, which are 462.41>282.34(Psychosine) and 467.4>287.4(d5-Psychosine). MassLynx 4.1 software was used for data analysis.

For galactosylceramide, galactosylceramide species were separated from glucosylceramide species by SFC-MS/MS on equipment consisting of a Waters UPC2 system coupled to a Thermo Scientific Quantum Access Max triple quadrupole mass spectrometer, also equipped with an ESI source. The instrument was operated in positive ion mode using multiple reaction monitoring. Chromatographic separations are obtained utilizing carbon dioxide gas and ammonium formate, formic acid in the methanol mobile phase, as previously described (21, 22, 23). The sphingolipid levels were expressed as picomoles per milligram (mg) of protein or dry-weight tissue (24).

### Quantification and statistical analysis

Statistical analyses were performed with GraphPad Prism version. 10.4.1(532) (GraphPad Software, LLC). Multiple comparisons were performed to assess statistical significance. When uniform variance of the results was identified by Bartlett’s analysis (*P* < 0.05), one-way analysis of variance (ANOVA) was used to test for statistically significant differences. When significant differences (*P* < 0.05) were identified, the results were further analyzed by Dunnett’s or the Tukey–Kramer multiple range test to determine the significance of differences between the groups. Where uniform variance of the results was not identified, non-parametric multiple comparisons were performed—after confirming significant differences (*P* < 0.05) using Kruskal–Wallis analysis, the differences were examined by applying Dunn’s multiple comparison test. Two-way ANOVA was performed to evaluate statistical significance. When significant differences (*P* < 0.05) were identified, the data were further analyzed using Dunnett’s multiple comparison test. Figure legends indicate sample sizes, and significance was defined as *P-*value∗<0.05, *p*∗∗<0.01, *p*∗∗∗<0.001, *p*∗∗∗∗<0.0001, ns: non-significant. The data were replicated using technical and biological replicates with a minimum of 3. The figure legends and supplementary information contain the information.

## Results

### MALDI method development for HexSP and HexCer

The study aims to apply a MALDI IMS method to examine the spatial biodistribution of HexSP and HexCer (d18:1/18:0) in the brain, correlating with the white matter demyelination process in GLD. To achieve this goal, the Twitcher mouse (*Galc*^*twi/twi*^), a C57BL6J mouse carrying naturally occurring biallelic Galc variants well-characterized by the accumulation of galactosylceramide and psychosine in the brain, was used (25). The general experimental workflow included technical and biological replicates. For the initial experiments, sagittal brain sections.

**Fig. 1:**
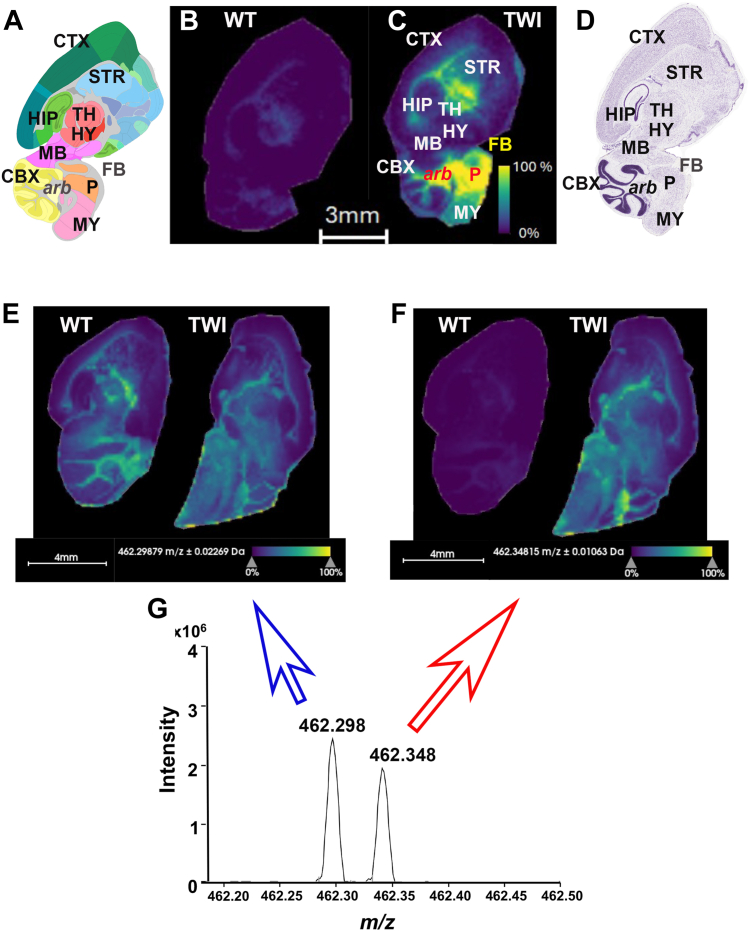
Visualization of key sphingolipid molecules in the neuropathogenesis of globoid-cell leukodystrophy (GLD) in mouse brain tissue sections. Anatomy of mouse brain sagittal section as reported in the Allen Brain Atlas (A), corresponding to the MALDI IMS of sagittal brain section from brain sections from wild type (WT; B) and Twitcher (TWI) *Galc*^*twi/twi*^ (C). The H&E-stained sagittal brain section exhibits the anatomy and orientation, and corresponding brain regions to the MALI IMS of the sagittal section of the TWI mouse. The brain regions including cerebral cortex (CTX), striatum (STR), hippocampus (HIP), thalamus (TH), hypothalamus (HY), midbrain (MB), cerebellum (CBX), *arbor vitae* (*arb*), pons (P), medulla (MY), and fiber tracts (FB) were successfully annotated in MALDI IMS (C) and corresponding H&E-stained (D) sagittal brain section images of Twi mouse using the Allen Brain Atlas (A). Most of the high-intensity signals from hexosylsphingosines (HexSP) colocalize with fiber tracts (FB) of the cerebrum, including the *arbor vitae* (*arb*) of the cerebellum and the pons (F) areas. The MALDI IMS image of the HexSP distribution in another sagittal section of the brains of Twi and Wt mice shows the corresponding HexSP spectra (F) and an isobar (E). G: The *m/z* charge spectrum shows the HexSP precursor ion at *m/z* 462.348 (F) and the corresponding isobar at *m/z* 462.298 (E). The lipid distribution corresponding to the isobaric lipid (blue arrow) showed comparable distribution in the TWI and WT sagittal brain sections (E). The HexSP distribution corresponding to the precursor ion (*m/z* 462.348; red arrow) showed distinct relative abundance and distribution of the sphingolipid across the brain sections of TWI and WT mice (F).

The MALDI profiling experiments were performed using authentic standards of psychosine and galactosylceramide to test and optimize their MALDI detection. For this purpose, different matrices, including 2,5-dihydroxybenzoic acid (DHB), 2′,6′-dihydroxyacetophenone (DHA), α-cyano-4-hydroxycinnamic acid, 1,5-diaminonaphthalene (DAN), 9-aminoacridine, and DAN were evaluated for the efficiency to facilitate ionization of these two glycosphingolipids in both positive and negative ion mode on the 7T solariX XR Fourier transform ion cyclotron resonance (FT-ICR) mass spectrometer. Among the tested matrices, DHA showed the best sensitivity of the psychosine standard and HexSP in positive ion mode (Fig. 1B, C), and it was identified using high-resolution accurate mass measurements (1.1 ppm). Regarding spatial resolution, the images of HexSP were acquired with a pixel spacing of 150 μm in both the *x* and *y* dimensions using a ∼135 μm diameter laser beam (Smartbeam II Nd:YAG laser system, 2 Hz, 355 nm). MALDI IMS of TWI brain sagittal sections show a remarkably higher HexSP signal in the fiber tract white matter areas, including the *corpus callosum,* cerebellum *arbor vitae*, and pons (Fig. 1A, C, D). The data for HexSP were collected from *m/z* 300 to 1,000 using a 0.3670-s time-domain transient length, resulting in a mass resolving power of ∼40,000 at *m/z* 426. Regarding ion image visualization, the resulting ion images of HexSP in the mouse brain sections were visualized using SCiLS (Bruker Daltonics) and displayed using a ±0.03 Da mass window without normalization. Interestingly, in contrast with the differences found in the HexSP distribution (*m/z* 462.348; Fig.1F, G), a corresponding isobaric lipid present at *m/z* 462.298 (Fig. 1G) revealed lipid distribution comparable in both the brain sagittal sections of the TWI and age-matched WT (Fig. 1E). For the galactosylceramide standard and HexCer, after testing the matrices mentioned above, DAN showed optimal ionization in negative ion mode. A pixel spacing of 150 μm in both the *x* and y dimensions was used with a ∼135 μm diameter laser beam, and HexCer data were collected from *m/z* 247 to 1,000 using a 2.4117-s time-domain transient length, resulting in a mass resolving power of ∼170,000 at *m/z* 726. HexCer (d18:1/18:0) was identified using high-resolution accurate mass measurements (0.21 ppm). The resulting ion images were visualized using SCiLS, and images were displayed using a ± 0.003 Da mass window without normalization.

More relevant and crucial than the matrix selection, the adoption of the CASI, aimed at enriching the HexSP and HexCer mass ranges of interest, was critical for improving signal intensity compared to full-scan measurements (supplemental Figs. S2 and S3). (17, 18). For HexSP, CASI allowed a mass resolving power of ∼40,000 at *m/z* 430 at data acquisition ranging from *m/z* 300 to 1,000 (supplemental Fig. S2). For HexCer, the CASI method generated a mass resolving power of ∼170,000 at m/z 726, when acquiring data ranging from *m/z* 250 to 1,000 (supplemental Fig. S3).

**Fig. 2:**
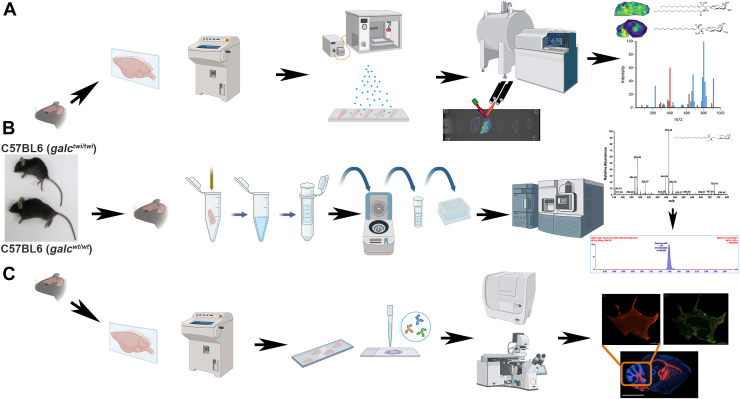
Experimental workflow of MALDI IMS and validation studies. Mouse brains were harvested, snap-frozen, and stored at −80 c (Materials and Methods). Subsequently, the harvested brains underwent 3 experimental procedures. A: Brains underwent cryo-section on a sagittal orientation and mounted onto indium tin oxide (ITO)-coated conductive glass slides and the chemical matrix, 2′,6′-dihydroxyacetophenone (DHA) for hexosylsphingosines (HexSP), and 1,5-diaminonaphthalene (DAN) for monohexosylceramide (HexCer). The matrix solutions were sprayed using a robotic sprayer. MALDI IMS of the two sphingolipids was performed at 150 μm pixel spacing in positive (HexSP) and negative (HexCer) ion modes. Structures of two Krabbe disease-relevant HexCer (galactosylceramide, top) and HexSP (psychosine, lower) are depicted next to their respective MALDI IMS ion images from brain sagittal sections from the Twitcher (TWI). B: For mass spectrometry-based lipidomics experiments, one cerebrum hemisphere from the same harvested brains was subject to lipid extraction following sample cleaning and loading to an injection plate, followed by liquid chromatography−tandem mass spectrometry (LC-MS/MS) MRM-based quantification of psychosine and galactosylceramide levels. C: Finally, the other cerebrum hemisphere also underwent cryosections, was mounted on frost slides, and followed fixation, permeabilization, and immunohistochemistry protocols using specific antibodies to myelin (MPB), neurofilament (NFH), astrocyte (GFAP), and microglial (Iba1) markers.

To validate the MALDI IMS method, a workflow was set to confirm the HexSP and HexCer semi-quantitative levels using traditional LC-MS/MS for psychosine and glucosylsphingosine, as well as galactosylceramide and glucosylceramide, along with immunohistochemistry assays (Fig. 2).

### Natural history of HexSP and HexCer brain spatial distribution

**Fig. 3:**
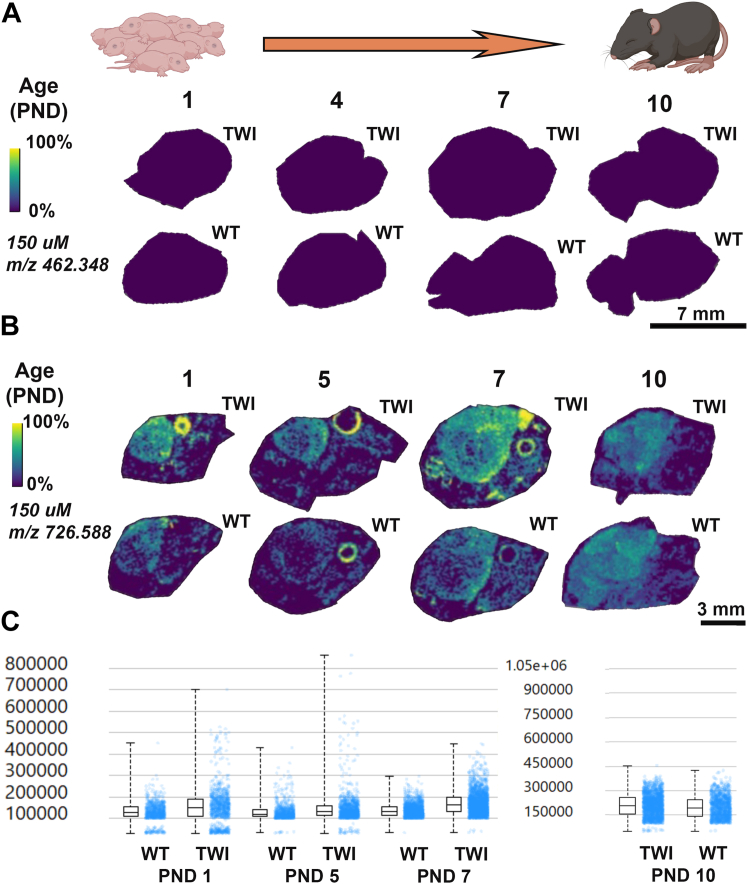
MALDI IMS images of HexSP and HexCer distribution in the sagittal brain sections of Twitcher (TWI) and wild type (WT) from postnatal days (PND) 1 to 10. A: MALDI IMS images of hexosylsphingosine (HexSP) from brain sagittal sections of TWI and WT at PND 1, 4, 7, and 10. B: MALDI IMS images of monohexosylceramide (HexCer) from brain sagittal sections of TWI and WT at PND 1, 5, 7, and 10. Localization profiles were generated at a 150 μm pixel spacing for both sphingolipids. C: Intensity box plots were created to show the relative abundance of HexCer in TWI and WT brain sagittal sections. Since the HexSP levels in the MALDI IMS images were substantially low in both TWI and WT brain sections (A), no intensity box blots were generated. The yellow color indicates the maximum signal intensity (100%), while the dark blue/black colors denote the minimum signal intensity (0%) of the ion selected. Scale bars are shown in the respective groups of brain sagittal sections for MALDI IMS for HexSP (A) and HexCer (B).

To investigate the spatial localization of the detected accumulated glycosphingolipids elevated in GLD in specific brain regions, ion intensity maps were generated for corresponding *m/z* values of the two sphingolipids. MALDI IMS was performed in positive ion mode for HexSP and negative ion mode for HexCer (d18:1/18:0). In the first ten postnatal days (PND) of life of both TWI and WT mice, minimal HexSP is detected in the brain at different ages. No difference in the HexSP distribution between TWI and WT is noticeable (Fig. 3A). The color scale depicts the relative intensity for HexSP (Fig. 3A) and HexCer (Fig. 3B). The localization profiles for HexSP and HexCer were generated at a spatial resolution of 150 μm (Fig. 3) at different ages ranging from 1 to 10 days. A representative of 3 mice between PND 1–10 is shown in Fig. 3. The detection of HexCer is more robust than that of HexSP at these early time points (Fig. 3B). Interestingly, HexCer is shown to have a slightly elevated and broader distribution in the middle brain of the first 10 days, with higher levels in the TWI mice, where compared to the WT (Fig. 3C). Since no quantifiable signal was detected for HexSP between PND 1–10 in the TWI and neither WT brains, no intensity box plots of HexSP were generated.

**Fig. 4:**
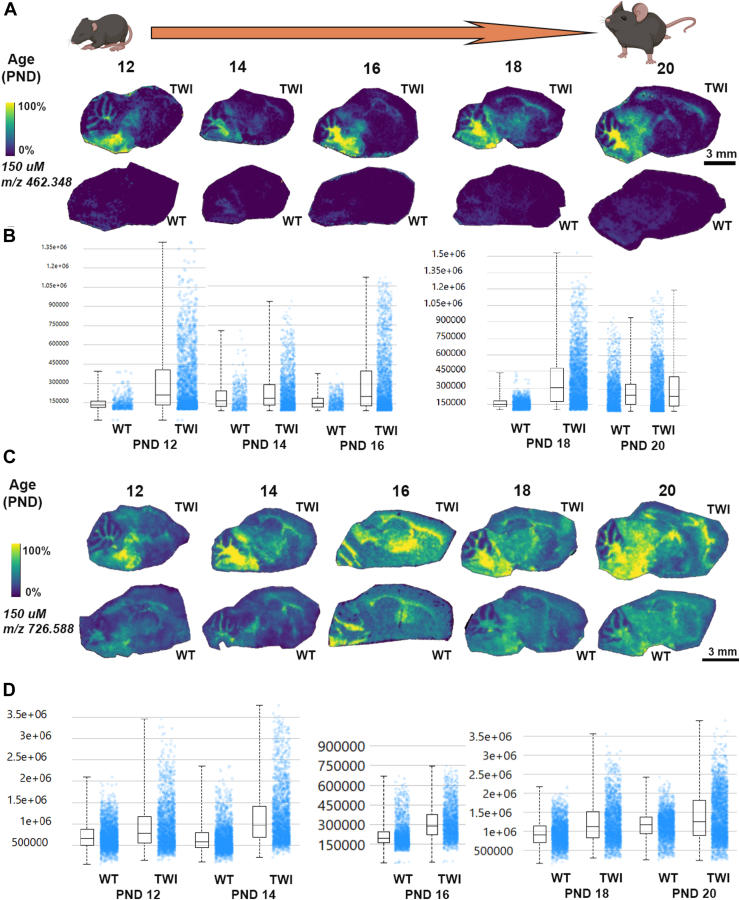
MALDI IMS images of HexSP and HexCer distribution in the sagittal brain sections of Twitcher (TWI) and wild type (WT) from postnatal days (PND) 12 to 20. A: MALDI IMS images of hexosylsphingosine (HexSP) from brain sagittal sections of TWI and WT at PND 12, 14, 18, and 20. B: The intensity box plots show the relative abundance of HexSP in TWI and WT brain sagittal sections. C: MALDI IMS images of monohexosylceramide (HexCer) from brain sagittal sections of TWI and WT at PND 12, 14, 18, and 20. Localization profiles were generated at a 150 μm pixel spacing for both sphingolipids. D: The intensity box plots were created to show the relative abundances of HexCer in TWI and WT brain sagittal sections. The yellow color indicates the maximum signal intensity (100%), while the black colors denote the minimum signal intensity (0%) of the ion selected. Scale bars are shown in the respective groups of brain sagittal sections for MALDI IMS for HexSP (A) and HexCer (C).

On the other hand, from PND 12, significant differences in the HexSP distribution are observed, as shown by intensity box plots comparing the relative HexSP abundances in TWI and WT brain tissue sections (Fig. 4A, B). In the color scale, the yellow signal represents higher HexSP levels located mainly in the fiber tracts regions of the cerebrum and cerebellum of brain sections of mice aged PND 12 to 20 (Fig. 4A). Between PND 10 and 20, the TWI mice show higher levels of HexSP with a broader distribution across the cerebrum and cerebellum, which intensifies as the mice age (Fig. 4A). In mice of the same age period, MALDI IMS showed that the HexCer distribution in the brain sections also follows the white matter in the fiber tracts regions; however, significant signals of the HexCer are also observed in the WT controls (Fig. 4C).

**Fig. 5:**
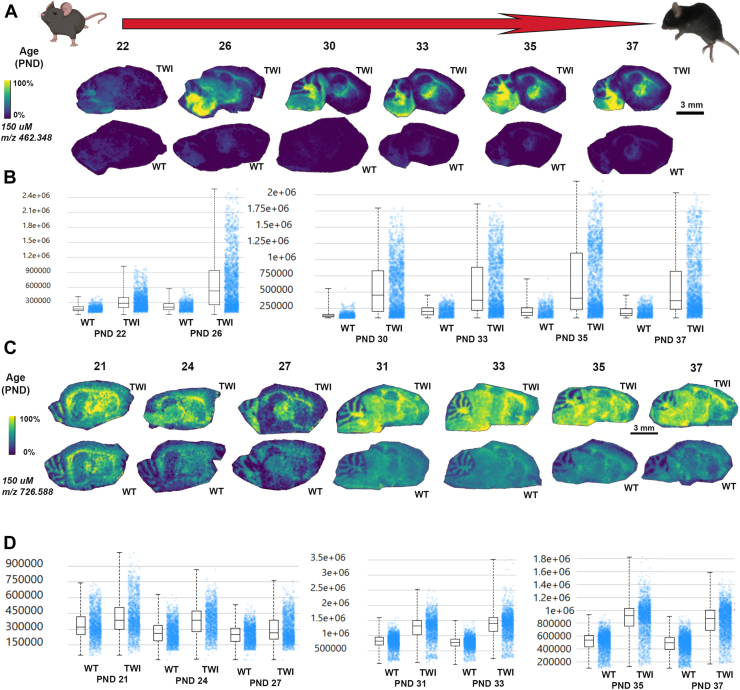
MALDI IMS images of HexSP and HexCer distribution in the sagittal brain sections of Twitcher (TWI) and wild type (WT) from postnatal days (PND) 22 to 37. A: MALDI IMS images of hexosylsphingosines (HexSP) from brain sagittal sections of TWI and WT at PND 22, 26, 30, 33, 35, and 37. B: The intensity box plots show the relative abundance of HexSP in TWI and WT brain sagittal sections. C: MALDI IMS images of monohexosylceramides (HexCer) from brain sagittal sections of TWI and WT at PND 21, 24, 27, 31, 33, 35, and 37. Localization profiles were generated at a 150 μm pixel spacing for the two sphingolipids. D: The intensity box plots show the relative abundance of HexCer in TWI and WT brain sagittal sections. The yellow color indicates the maximum signal intensity (100%), while the black colors denote the minimum signal intensity (0%) of the ion selected. Scale bars are shown in the respective groups of brain sagittal sections for MALDI IMS for HexSP (A) and HexCer (C).

From ages PND 22 to 37, the progressive pattern of increased intensity of HexSP signals is noticeable, with a higher intensity in the supra-tentorial areas of the TWI brain (Fig. 5A). The brain sections from the WT mice show nearly no detectable HexSP signal, as noted in the lowest signal intensity (dark blue) (Fig.5A, B). In terms of HexCer, significant but less extended differences are noticeable between the TWI and the WT brain sections (Fig. 5C). However, as the TWI becomes progressively physically debilitated with pronounced neurological symptoms, increased intensity and broader distribution of the HexCer intensity signals are observed in the TWI brain, especially in the PND 35 and 37, which is also indicated by the intensity box blots normalized to total ion count (TIC) (Fig. 5D).

### LC-MS/MS HexCer and HexSP brain spatial distribution

**Fig. 6:**
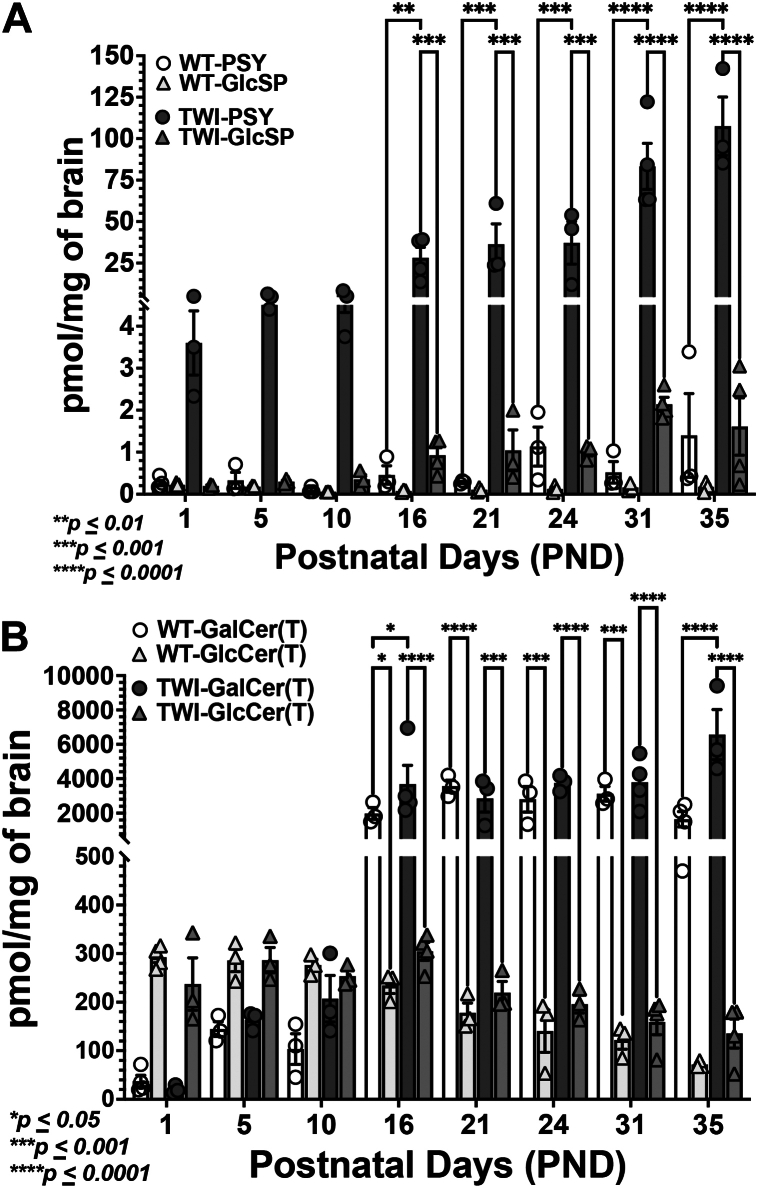
LC-MS/MS measurement of psychosine, glucosylsphingosine, total galactosylceramides, and total glucosylceramides from brain specimens from the Twitcher (TWI) and wild-type (WT) mice used for MALDI IMS. A: Levels of psychosine (PSY) and glucosylsphingosine (GlcSP) measured by liquid chromatography-tandem mass spectrometry (LC-MS/MS) from the brain sagittal sections of TWI and WT undergoing the MALDI IMS across the different ages of their life span are shown in the histogram (each postnatal day, PND, represents a mean of 3 mice). B: Levels of total galactosylceramides, GalCer(T), and total glucosylceramides, GluCer (T), measured by LC-MS/MS from the brain sagittal sections of TWI and WT undergoing the MALDI IMS across the different ages of their life span are shown in the histogram (each PND represents a mean of 3 mice). Levels of different hexosylsphingosines and hexosylceramides (pmols/mg of prot) were calculated using specific deuterated-labeled internal standards and a calibration standard curve. The specific levels of GalCer and GluCer species are depicted in supplemental Fig. S1.

To confirm the intensity signals and differences observed by MALDI IMS, we performed quantitative measurements of the HexSPs: psychosine and glucosylsphingosine; and HexCer: galactosylceramide and glucosylsphingosine, via LC-MS/MS from selected brain specimens previously analyzed. In the TWI mouse group secondary to the Galc deficiency, the elevated psychosine levels, which are significantly higher than the isobaric glucosylsphingosine, correlate with the HexSP IMS ion images, showing a progressive trend of psychosine levels (Fig. 6A). Thus, the large majority of the HexSP signals in the MALDI IMS of the brain of the TWI mouse groups are contributed by psychosine (galactosylsphingosine). The MALDI IMS method was able to detect HexSP levels as low as ∼10–20 pmol/mg of protein of psychosine as quantified by LC-MS/MS (Fig. 6A). The higher sensitivity of LC-MS/MS revealed significantly elevated psychosine levels in the brain specimens from TWI brain from PND 1, when the MALDI IMS is unable to detect differences in HexSP between TWI and WT mouse groups (Fig. 3).

**Supp Fig 1:**
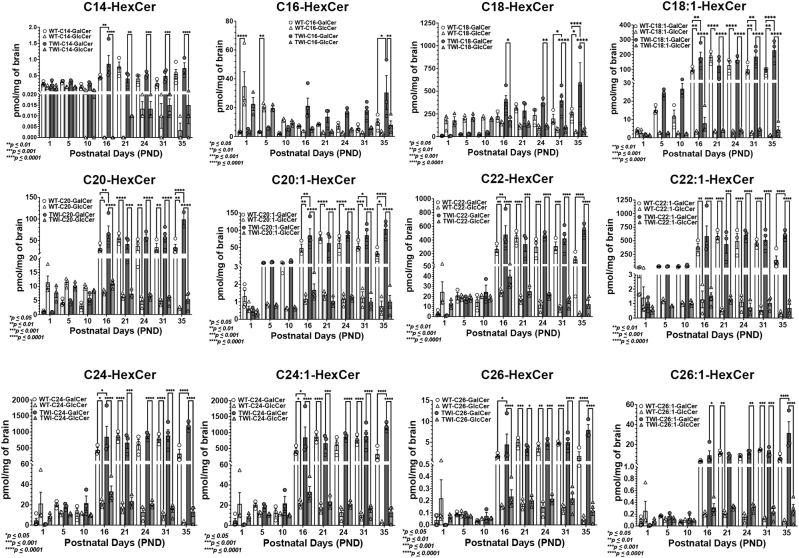


Regarding the galactosylceramide, another Galc primary substrate, significant but minor differences between the levels of this glycosphingolipid are observed in the brain specimens from TWI and WT mice throughout their lifespans (Fig. 6B). The LC-MS/MS measurements showed that, after PND10, the total galactosylceramide, GalCer(T), is significantly higher than total glucosylceramides, GlcCer(T), in both TWI and WT mouse groups (Fig. 6B). The same pattern was noted across different GalCer and GlucCer species, as shown in supplemental Fig. S1. Similarly, consistent with the LC-MS/MS analysis, the MALDI IMS showed comparable minor differences, but higher HexCer levels throughout the white matter regions of the brain specimens of the TWI mice group in comparison with the WT (Figs. 3, 4, and 5).

### Brain MALDI IMS and immunohistochemistry correlation

**Fig. 7:**
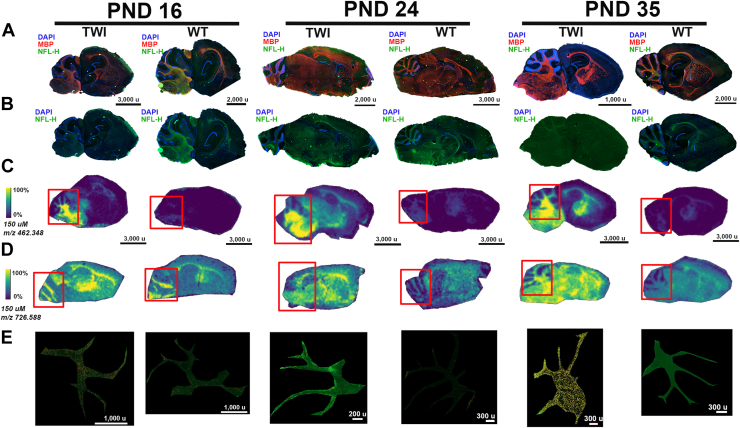
Brain MALDI IMS and Immunohistochemistry Correlation. A: Brain sagittal sections from Twitcher (TWI) and wild type (WT) mice at postnatal days (PND) 16, 24, and 35 were evaluated for specific immunostainings for myelin-basic protein (MPB), neurofilament H (NFL-H), and GFAP. (A) The brain sagittal sections of TWI and WT are stained with MBP, NLF-H, and DAPI. B: The brain sagittal sections of TWI and WT are stained with DAPI and NLF-H, showing a decreased neurofilament stain H (NFL-H) immunostained in the brain specimens from the TWI over the PNDs. The MALDI IMS images for hexosylsphingosine (C) and monohexosylceramide (D) correspond to the brain sagittal sections of TWI and WT undergoing immunostaining. Over the MALDI IMS imaging, a red square area indicates the selected *arbor vitae* area of the cerebellum from the GFAP staining (E). In the selected *arbor vitae,* GFAP-positive astrocytes are yellow-marked and more pronounced in the TWI brain sections.

Since HexSP showed a clear correlation with the biodistribution of the brain, three different pairs of brain sections from TWI and WT mice at PND 16, 24, and 35, which underwent MALDI IMS, were also analyzed for the specific myelin (MBP), neurofilament (NFL-H), and astrocyte (GFAP) markers (Fig. 7). Across different ages of TWI, although still present, an irregular and inconsistent distribution of the myelin-basic protein (MBP) is noticeable in the brain sections of TWI mice. In PND 16 through 35, irregularity of the MPB distribution, with areas with absent staining (Fig. 7A), was primarily noted in the *arbor vitae* (white matter core region) of the cerebellum, where the highest intensity signal of HexSP is noticeable (Fig. 7C). Interestingly, in the brain sections of the TWI mice, the neurofilament H (NFL-H, heavy chain) integrity is disrupted throughout the fiber tracts regions and showed a reduced signal when compared to the sections of age-matched WT mice (Fig. 7B). In the cerebellum's *arbor vitae* (white matter core region) from the TWI brain sections (Fig. 7C, E – red squares), substantial astrogliosis indicates the inflammatory process (Fig. 7E), which is more pronounced at PND 35 when advanced disease is observed (Fig. 7D).

## Discussion

In this study, we developed a novel MALDI IMS method to examine the spatial biodistribution of HexSP and HexCer in the brain, correlating with the white matter demyelination process in GLD. The Twitcher mouse (TWI, *Galc*^*twi/twi*^), the naturally occurring C57BL6J mouse GLD model with biallelic nonsense *Galc* variants, (25) was used to achieve this goal. For the initial experiments, sagittal brain sections from 35-day-old male and female TWI, which show the highest levels of psychosine and galactosylceramide in the CNS and PNS, were used, with age-matched WT C57BL6 mice used for the MALDI IMS experiments.

**Supp Fig 2:**
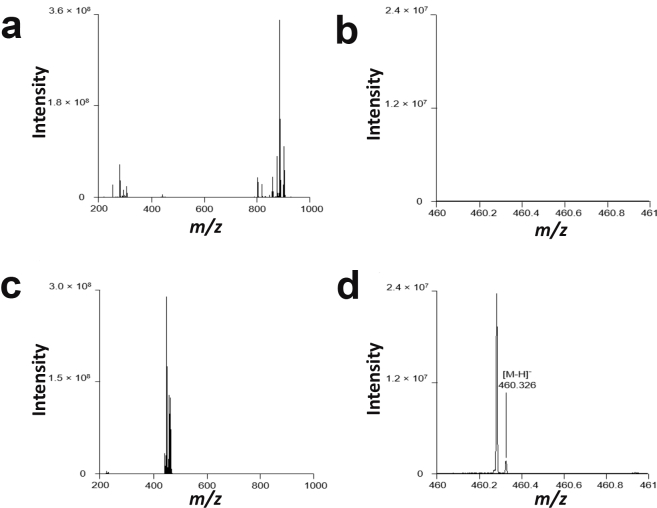


The MALDI profiling experiments were performed on different matrices to examine the efficiency of facilitating the ionization of these two types of glycosphingolipids in both positive and negative ion modes. As described earlier, the DHA generated the best sensitivity of the HexSP in positive ion mode (Fig.1B, C), using high-resolution accurate mass measurements (1.1 ppm). In terms of the brain regions, the MALDI IMS of TWI brain sagittal sections show a remarkably higher HexSP signal in the fiber tract white matter areas, including the *corpus callosum,* cerebellum *arbor vitae*, and pons (Fig. 1A, C, D). To ensure the specificity of the MALDI IMS signal for HexSP (*m/z* 462.348; Fig. 1F, G), a corresponding isobaric lipid present at *m/z* 462.298 (Fig. 1G) revealed lipid distribution comparable in both the brain sagittal sections of the TWI and age-matched WT (Fig. 1E). Regarding the HexCer sphingolipid, the DAN showed optimal ionization in negative ion mode. A pixel spacing of 150 μm in both the *x* and *y* dimensions with a ∼135 μm-diameter laser beam was used for HexCer, whose data were collected between *m/z* 247 to 1,000 using a 2.4117-s time-domain transient length, resulting in a mass resolving power of ∼170,000 at *m/z* 726. The HexCer (d18:1/18:0) was identified using high-resolution accurate mass measurements (0.21 ppm). Beyond matrix selection for the MALDI IMS method, adopting the CASI mode increases the sensitivity of both HexSP and HexCer analytes (supplemental Figs. S2 and S3).

With the MALDI IMS developed method targeting 2 types of sphingolipids relevant to GLD, we designed and followed the workflow depicted in Figure 2 to confirm the HexSP and HexCer semi-quantitative levels using traditional LC-MS/MS and immunohistochemistry. To investigate the spatial distribution in specific brain regions of the elevated glycosphingolipids in GLD, ion intensity maps were generated for corresponding *m/z* values of the two sphingolipids. The MALDI IMS was performed in positive ion mode for HexSP and negative ion mode for HexCer (d18:1/18:0). To determine the natural history of the two neuropathogenic sphingolipids in GLD, we set up an examination of TWI and WT mice in several postnatal days (PND) of age ranging from 1 to 37 days of life. A representative of 3 mice between PND 1–10 is shown in Figure 3. Using the MALDI IMS method, no differences in the HexSP detected signals in the brain between TWI and WT mice were noticeable in the first 10 PNDs (Fig. 3A). The localization profiles for HexSP and HexCer were generated at a spatial resolution of 150 μm (Fig. 3) at different ages ranging from PND 1 to 10. The detection of HexCer is more robust than that of HexSP at these early age time points (Fig. 3B). Interestingly, in the first 10 days of age, HexCer iis slightly elevated with a broader distribution in the middle brain of the TWI mice compared to HexSP (Fig. 3C). Since no quantifiable signal was detected for HexSP between PND 1–10 in the TWI and neither WT, no intensity box plots of psychosine were generated. On the other hand, from PND 12 onward, significant increases in the HexSP signals and distribution in the TWI brain were observed and intensity box plots compared to the WT brain tissue sections are depicted (Fig.4A, B). The highest HexSP levels were located mainly in the fiber tract regions of the cerebrum and cerebellum of brain sections of mice aged PND 12 to 20 (Fig. 4A). From the PND 10 to 20, the TWI mice show elevated HexSP with a broader distribution across white matter regions of the cerebrum and cerebellum in comparison to the age-matched WT (Fig. 4A). Also, in the PND 10–20 period, the MALDI IMS signals from HexCer showed increased distribution in the TWI brain sections, which also follows the white matter in the fiber tract regions. Interestingly, significant signals of the HexCer are also observed in the WT age-matched controls (Fig. 4C). This observation is likely due to the essential role of the HexCer, mostly GalCer, in myelin stability, and also being the major glycosphingolipid in compact myelin (26). In addition, higher HexCer intensity signals are observed in brain sagittal sections from older TWI and controls (Fig. 4C). At the age period from PND 22 to 37, a progressive augmentation of the intensity of HexSP signals is noticeable, with a higher intensity in the supra-tentorial areas of the TWI brain (Fig. 5A). In comparison, the age-matched WT mice show nearly no detectable HexSP signals, as (dark blue; Fig.5A, B). Regarding the HexCer signals, significant but less evident differences were observed between the TWI and WT brain sections (Fig. 5C). Nonetheless, as the neurodegenerative disease progresses with pronounced neurological symptoms in the TWI mice, increased intensity and broader distribution of the HexCer intensity signals are observed in their brain, especially in the PND 35 and 37. The intensity box blots corroborate those observations in the MALDI IMS signals normalized to TIC (Fig. 5D).

One of the limitations of the MALDI IMS method developed is its inability to distinguish the psychosine (galactosylsphingosine) from its isomer glucosylsphingosine (GlcSP) as well as the galactosylceramide (GalCer) from glucosylceramide (GlcCer). For this reason, the HexSP and HexCer nomenclature was adopted to describe the signals generated by the novel MALDI IMS developed. For method validation, quantitative measurements of psychosine, GlcSP, GalCer, and GlcCer were performed by LC-MS/MS on selected brain specimens that underwent MALDI IMS analysis. The psychosine levels, determined by LC-MS/MS, correlated with the MALDI IMS ion images, showing a progressive trend of psychosine levels (Fig. 6A). Actually, the measurements of psychosine and GlcSP by LC-MS/MS from TWI brain specimens showed that psychosine significantly contributes to over 94% of the total HexSP signals detected by MALDI IMS (Fig. 6A). Therefore, the majority, or nearly the entire HexSP signals in TWI are contributed by psychosine (Figs. 4A, 5A, and 6A). The brain specimens from WT mice, regardless of age, showed no HexSP signal in the MALDI IMS method (Figs. 3A, 4A, and 5A). In addition, the LC-MS/MS, as expected, supersedes the sensitivity of the MALDI IMS method and was able to detect psychosine levels as low as ∼10–20 pmol/mg of protein (Fig. 6A). From PND1, the TWI mice brain specimens showed significantly higher psychosine levels from age-matched WT mice brains (Fig. 6A). Given that the TWI recapitulates the infantile, early-onset clinical form of GLD, this observation underscores the need for early diagnosis and immediate initiation of therapeutic management to arrest further cytotoxic enhancements of psychosine in the CNS and PNS. Interestingly, a slight increase in low levels of psychosine is observed in WT brain specimens at PND24 and PND35 (Fig. 6A), indicating the glycosphingolipid physiological role in the myelination process. In terms of the GalCer, also a Galc primary substrate, significant but minor differences in the brain specimens from TWI and WT mice are observed across their lifespan (Fig. 6B). Consistent with the LC-MS/MS analysis, the HexCer signals (mostly represented by galactosylceramide) in the MALDI IMS showed comparable minor differences, but higher GalCer levels throughout the white matter regions of the brain specimens of the TWI mice compared to the WT at PND 35 (Figs. 4A, 5A, and 6A). GalCer is the most abundant sphingolipid, accounting for approximately 20% of the myelin lipids in the CNS (27, 28). GalCer species are present in the myelin bilayer and preferentially consist of a galactose constituting the head group, a sphingosine-based backbone, and a very long-chain fatty acid tail group (Fig. 2A). GalCer species are extremely hydrophobic molecules, generating intermolecular hydrophobic forces contributing to myelin membrane “zippering” (26). For these reasons, being the major glycosphingolipid component of myelin, the levels of total GalCer in the brain specimens of TWI did not differ remarkably from those of the WT in MALDI IMS (Fig. 3, Fig. 4, Fig. 5), which is corroborated by the LC-MS/MS analysis (Fig. 6B). In addition, the LC-MS/MS analysis of the brain specimens undergoing MALDI IMS showed that GalCer(T) significantly contribute to 92%–97% of the HexCer signals detected in the brains of TWI and WT mice at PND16 and older (Fig. 6B). Therefore, the HexCer signals in MALDI IMS (Figs. 3C, 4C, 5C) correlate to the LC-MS/MS analysis through which measurable GalcCer(T) levels are observed in WT brain specimens (Fig. 6B). Interestingly, at PND 1-5, in both TWI and WT brain specimens, GlcCer(T) predominates over GalCer(T) (Fig. 6B). At PND 10, in TWI brain specimens, GalCer(T) and GlcCer(T) proportions are similar, likely secondary to the progressive increase of GalCer levels in the setting of enzymatic Galc deficiency (Fig. 6B). Whereas, in the WT brain specimens, GlcCer(T) still remains higher than GalCer(T), contributing ∼72% of HexCer (Fig. 6B). The acylation of the increased levels of GalCer by the lysosomal acid ceramidase can contribute to the reduced differences of the GalCer(T) levels in the TWI, given the Galc deficiency (10). The complete analysis of the different GalCer and GlcCer species in the brain specimens of the TWI and WT mice at different PNDs is depicted in the supplemental Fig. S1.

To further validate the MALDI IMS method, three different pairs of brain sections from TWI and WT at PND 16, 24, and 35 that underwent MALDI IMS analysis were selected for immunohistochemistry assay for the specific myelin (MBP), neurofilament (NFL-H), and astrocyte (GFAP) markers (Fig. 7). The MBP signal showed an irregular and inconsistent distribution—some areas with absent MPB-staining—in brain sections of the TWI mice at different stages (Fig. 7A). The irregularity was particularly noticeable in the cerebellum *arbor vitae* (white matter core region), which correlates with the highest levels of psychosine ascertained by the MALDI IMS method (Fig. 7C). In the same brain sections of the TWI mice, NFL-H showed compromised integrity in the fiber tract cerebrum regions and reduced signals in comparison to age-matched WT mice (Fig. 7B), indicating the psychosine-mediated dephosphorylation of the neurofilaments as previously described (29). In the cerebellum's *arbor vitae* (white matter core region) from the TWI brain sections, substantial astrogliosis indicates the inflammatory process, which is more pronounced at PND 35 when advanced disease is observed (Fig. 7D). The TWI is widely considered the classical murine model for GLD and has been extensively characterized regarding the neurobehavioral aspects (25, 30, 31, 32, 33, 34), including the correlation with the sphingolipid levels and histopathology (8, 10, 25, 35, 36, 37, 38, 39, 40). Several of the MALDI IMS patterns of the HexSP and HexCer spatial distribution can be correlated to the described murine neurobehavioral disease stages previously reported (25, 30, 31, 32, 33, 34).

Another limitation of our current work is that we have focused on the two major primary glycosphingolipid substrates of Galc, which are known to be involved in the neuropathogenesis of GLD (5, 9, 10, 29, 41, 42, 43, 44, 45). Future work is required to determine whether impairment and correlations of disturbances of other sphingolipids are related to sphingosine and ceramides. Furthermore, it would be interesting to visualize alterations in proteins associated with myelin across different brain regions of the TWI mouse using MALDI IMS.

Using a newly developed MALDI IMS, the brain mapping and visualization of the two neuropathogenic sphingolipids in the classical murine Krabbe disease model is demonstrated here. The MALDI IMS allows for the first-time determination of the spatial and relative quantitation of HexSP and HexCer (d18:1/18:0) in the brains of the TWI and age-matched WT mice. The LC-MS/MS measurements of HexSP and HexCer levels of the identical specimens used in the MALDI IMS validated the intensity signals of these sphingolipids detected in the brains of TWI and WT mice throughout their lifespan. The wide differences in the HexSP MALDI IMS signals between the TWI and WT brain sections correlated with the LC-MS/MS measurements of psychosine. HexCer MALDI IMS signals showed lower differences between the brain specimens from the TWI and age-matched WT, which corresponds to the GalCer measurements by LC-MS/MS. In addition, the spatial location of the high signals of HexSP detected by MALDI IMS in the TWI brain correlated with areas of the white matter where irregular MPB signals, disrupted NFL-H signals, and increased astrocyte recruitment are observed. Paradoxically, the limitation of the proposed MALDI IMS approach in resolving galactosyl and glucosyl forms of HexSP and HexCer can also be advantageous for its application to other inborn lysosomal disorders, such as glucocerebrosidase deficiency (Gaucher disease), as the spatial distribution and relative quantification of GlcSP and GlcCer in the CNS are also relevant.

Overall, the MALDI IMS method showed a robust free-label tool to examine both the spatial distribution and abundance of two pathogenic sphingolipids in Krabbe disease, providing information on emerging therapeutic interventions, primarily related to their CNS biodistribution and correlations with changes in neuropathology and neurobehavioral outcomes.

## Data availability

All data reported will be shared by the lead contact upon request. No original code is reported in this research article. Any additional information required to reanalyze the data reported in the research article is available from the lead contact upon request.

## Supplemental data

This article contains supplemental data.

## Conflict of interest

G. H. B. M. serves as a consultant for Takeda Pharm., Sanofi, and Chiesi.

## Figures and Tables

**Supp Fig 3:**