# Supplementary material for: Spatial and temporal brain biodistribution of neuropathogenic sphingolipids of Krabbe disease
Source: J Lipid Res. 2025 Dec 5;67(1):100960. doi: 10.1016/j.jlr.2025.100960 (PMC12819350; doi:10.1016/j.jlr.2025.100960)
Supplement: Supplemental data [file mmc1.docx]

**Supplementary Materials**

**Spatial and Temporal Brain Biodistribution of Neuropathogenic Sphingolipids of Krabbe Disease**

Tingting Yan *et al.*

**Corresponding Author Information**

*Gustavo H. B. Maegawa, M.D., Ph.D.

Columbia University Irving Medical Center

630 W168th St. – VP&S 9^th^ FL. – 9-401C

New York, NY 10032

United States

Phone 212-342-3647

Fax 212-305-9058

gm3025@cumc.columbia.edu

**The PDF file includes:**

Suppl.Fig.1 Legend

**Legends**

**Supp.Fig.1. Galactosylceramide (GalCer) and glucosylceramide (GalCer) species measurements from brain specimens from Twitcher (TWI) and age-matched wild-type (WT).**

**Supp.Fig.2. Analysis of hexosylsphingosine (HexSP) in Twitcher mouse (*Galc^twi/twi^*).** The full-scan acquisition mode of HexSP (**a, b**) and Continuous Accumulation of Selected Ions (CASI) mode using a 30 Da window centered at m/z 460 (**c, d**) are shown. Panels **b** & **c** are spectral magnifications of the analyte of interest, HexSP.

**Supp.Fig.3. Analysis of monohexosylceramide (HexCer) in Twitcher mice (*Galc^twi/twi^*).** The full-scan acquisition mode of HexCer (**a, b)** and Continuous Accumulation of Selected Ions (CASI) mode using a 10 Da window centered at m/z 729 (**c, d**) are shown. Panels **b** & **c** are spectral magnifications of the analyte of interest, HexCer.
